# A targeted in situ hybridization screen identifies putative seminal fluid proteins in a simultaneously hermaphroditic flatworm

**DOI:** 10.1186/s12862-018-1187-0

**Published:** 2018-05-30

**Authors:** Michael Weber, Julia Wunderer, Birgit Lengerer, Robert Pjeta, Marcelo Rodrigues, Lukas Schärer, Peter Ladurner, Steven A. Ramm

**Affiliations:** 1 Evolutionary Biology, Bielefeld University, Konsequenz 45, 33615 Bielefeld, Germany; 2 Institute of Zoology and Center of Molecular Biosciences Innsbruck, University of Innsbruck, Technikerstr. 25, A-6020 Innsbruck, Austria; 3 Evolutionary Biology, Zoological Institute, University of Basel, Vesalgasse 1, 4051 Basel, Switzerland; 4 Current address: School of Natural and Environmental Sciences, Ridley Building, Newcastle University, Newcastle upon Tyne, England NE1 7RU UK

**Keywords:** Seminal fluid, Flatworm, In situ hybridization, Prostate, Sex allocation, Sexual selection, Sperm competition, Sexual conflict, Allohormone

## Abstract

**Background:**

Along with sperm, in many taxa ejaculates also contain large numbers of seminal fluid proteins (SFPs). SFPs and sperm are transferred to the mating partner, where they are thought to play key roles in mediating post-mating sexual selection. They modulate the partner’s behavior and physiology in ways that influence the reproductive success of both partners, thus potentially leading to sexual conflict. Despite the presumed general functional and evolutionary significance of SFPs, their identification and characterization has to date focused on just a few animal groups, predominantly insects and mammals. Moreover, until now seminal fluid profiling has mainly focused on species with separate sexes. Here we report a comprehensive screen for putative SFPs in the simultaneously hermaphroditic flatworm *Macrostomum lignano*.

**Results:**

Based on existing transcriptomic data, we selected 150 transcripts known to be (a) predominantly expressed in the tail region of the worms, where the seminal fluid-producing prostate gland cells are located, and (b) differentially expressed in social environments differing in sperm competition level, strongly implying that they represent a phenotypically plastic aspect of male reproductive allocation in this species. For these SFP candidates, we then performed whole-mount in situ hybridization (ISH) experiments to characterize tissue-specific expression. In total, we identified 98 transcripts that exhibited prostate-specific expression, 76 of which we found to be expressed exclusively in the prostate gland cells; additional sites of expression for the remaining 22 included the testis or other gland cells. Bioinformatics analyses of the prostate-limited candidates revealed that at least 64 are predicted to be secretory proteins, making these especially strong candidates to be SFPs that are transferred during copulation.

**Conclusions:**

Our study represents a first comprehensive analysis using a combination of transcriptomic and ISH screen data to identify SFPs based on transcript expression in seminal fluid-producing tissues. We thereby extend the range of taxa for which seminal fluid has been characterized to a flatworm species with a sequenced genome and for which several methods such as antibody staining, transgenesis and RNA interference have been established. Our data provide a basis for testing the functional and evolutionary significance of SFPs.

**Electronic supplementary material:**

The online version of this article (10.1186/s12862-018-1187-0) contains supplementary material, which is available to authorized users.

## Background

During ejaculate transfer, sperm cells are often accompanied by a complex mixture of additional substances – known collectively as seminal fluid – that is produced in the male accessory reproductive glands. Some of these components play important roles in nourishing and activating the sperm themselves [1], whereas others act – either independently of, or in association with, sperm [2] – to influence subsequent female physiology and behaviour in various ways, all of which can affect the outcome of sperm competition [3]. For example, specific seminal fluid proteins (SFPs) are known to modulate the receptivity of the female, reduce female attractiveness to future potential mates, induce oviposition, change egg laying rate, increase egg production, stimulate egg maturation, change feeding behavior, and also increase female mortality rate [3–5]. Because all of these responses to SFPs likely affect male reproductive success, SFPs are important targets of sexual selection, likely explaining their rapid adaptive evolution [6–12]. Moreover, because seminal fluid can modulate female physiology and reproductive behavior in so many ways – possibly to the advantage of the seminal fluid-donating individual (i.e. the male, or more generally, the sperm donor), but not necessarily that of the seminal fluid-receiving individual (i.e. the female, or more generally, the sperm recipient) – SFP deposition is often expected to lead to sexual conflict [13], an additional factor likely driving rapid SFP evolution [14].

In species with separate sexes, there are numerous studies on seminal fluid composition and its functional effects. These mainly concern well-investigated species, mostly insects, including several Diptera [15–24], various Coleoptera [25–27], Orthoptera [8, 28], Lepidoptera [29, 30], Hymenoptera [31, 32] and Hemiptera [33, 34]. Besides insects, there are also studies on the characterization of seminal fluid in a few other taxa, most notably mammals, such as rodents [35, 36], livestock species [37–39] and humans [40, 41].

In simultaneous hermaphrodites (in which individuals possess both male and female sex functions at the same time), there are to date very few studies of seminal fluid. One example in hermaphrodites for the transfer of accessory gland substances is the shooting of so called love darts in land snails [42, 43], where the accessory gland substances are transferred via hypodermic injection [44, 45]. But for seminal fluid proteins that are transferred together with the sperm in the ejaculate, the proteins have partially been characterized only in a freshwater snail species [46]. This scarcity is unfortunate, because simultaneous hermaphroditism is a common reproductive mode throughout the animal kingdom [47], meaning that if we want to understand the effects of seminal fluid more generally, we also need to understand them in hermaphrodites [48]. Moreover, in hermaphrodites unique predictions about putative functions of seminal fluid have been made, since they might not only affect the female sex function of the mating partner (as in gonochorists), but also its male function (with possible knock-on effects for the female function, if this induces changes in resource allocation towards the female function [48–50]).

Seminal fluid effects on the partner’s male function in simultaneous hermaphrodites are not just a theoretical possibility: in the only simultaneous hermaphrodite in which seminal fluid has been studied in detail to date, the great pond snail *Lymnaea stagnalis*, effects on both the male and female functions have been observed. Several seminal fluid proteins were identified by HPLC and their functions examined by injecting specific proteins intravaginally [46, 51], revealing that seminal fluid receipt affects reproductive output in this species [46, 52–54]. More specifically, the receipt of one SFP (LyAcp10) had an effect on egg laying [51]. The intravaginal injection of two other SFPs (LyAcp8b and LyAcp5) resulted in a reduction of sperm transferred in a subsequent mating by the recipient in *L. stagnalis*, and as a result, in a decrease in the paternity success in subsequent matings as a male [46]. This study highlights that steering your partner away from its male function is a potentially adaptive strategy in simultaneous hermaphrodites [48].

Moreover, mating motivation in hermaphroditic individuals is likely driven more by the opportunity it provides to donate sperm to fertilize partners’ eggs rather than the opportunity to gain sperm to fertilize own eggs [49, 55]. This means two simultaneous hermaphrodites will often agree on mating, even with the possible disadvantage of receiving sperm (or of being unable to avoid receiving it because of reciprocal copulation). This fact, and the expected scarcity of pre-mating sexual selection [42, 56], leads to the assumption that there may be an enhanced role for postcopulatory sexual selection in hermaphrodites compared to gonochorists [55, 57]. To test the generality of these insights, and those gained to date in gonochorists, further hermaphroditic model systems for studying seminal fluid-mediated postcopulatory effects are clearly needed.

**Fig. 1:**
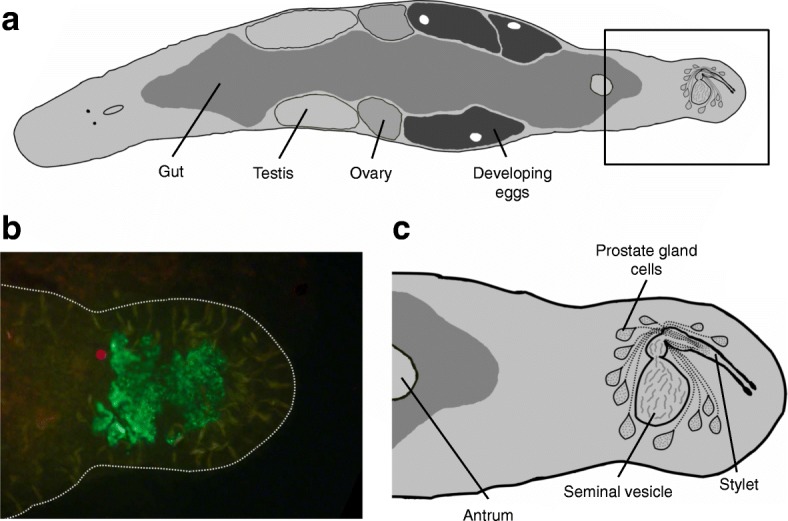
Schematic drawings and monoclonal antibody staining of specimens of *Macrostomum lignano.* **a** Habitus of *M. lignano* (ventral view); (**b**) Immunocytochemical staining of cytoplasmic content within the prostate glands with the mAB MPr-1 (the red dot is likely an auto fluorescence artifact); (**c**) posterior part of the animal with female genitalia (antrum) & male genitalia (comprising stylet, seminal vesicle and prostate gland cells, the latter two which can overlap)

In this study, we aimed to characterize seminal fluid in a previously unstudied hermaphroditic group. Our study organism is the free-living flatworm *Macrostomum lignano* (Lophotrochozoa: Platyhelminthes: Rhabditophora), which has recently emerged as a model organism in various other fields of biology [58, 59]. *M. lignano* is a simultaneous hermaphrodite with reciprocal copulation (i.e., during mating, each partner both donates and receives sperm and seminal fluid). This species has been intensively studied in relation to sex allocation theory [60–66]. Specifically, individuals can plastically allocate their resources towards the male or the female sex function [61, 65, 66] and we already know that they can plastically modify gene expression in several body regions, including their tail (the location of the SFP-producing prostate glands cells, Fig. 1) in response to changes in social group size (Ramm et al: Sex Allocation Plasticity on a Transcriptome Scale: Socially-Sensitive Gene Expression in the Hermaphroditic Flatworm *Macrostomum lignano*, submitted), which reflects mating group size (i.e. the number of mating partners plus one) [61, 65, 67]. This result is expected according to the mating group size model [68, 69], which predicts that the optimal investment in male function is low in the absence of sperm competition but, as mating group size increases, sperm competition drives the optimal male allocation up.

Until now, seminal fluid is not well studied in the genus *Macrostomum*, but recent results in *M. lignan*o both hint at the potential for seminal fluid-mediated effects and provide a basis on which to begin to identify individual seminal fluid components. Regarding potential seminal fluid functions, Marie-Orleach et al. [70] found that individuals mated to a virgin partner exhibit fewer instances of a so-called ‘suck behavior’, compared to individuals mated to a sexually experienced partner. This suck behavior is a post-copulatory behavior that is thought to be involved in removing ejaculate components received during copulation [71–73]. Marie-Orleach et al. [70] therefore concluded that seminal fluid could potentially inhibit the suck behavior, based on the assumption that virgin individuals are likely to transfer greater amounts of seminal fluid to their mating partners during copulation (because they have more already-produced seminal fluid in storage prior to copulation, as measured by how visually prominent the prostate gland cells appear in vivo in these transparent worms). In a second study, Marie-Orleach et al. [74] sough to partition variance in reproductive success via the male sex function into its component parts, finding that variance in male reproductive success mainly arises from sperm transfer success and not from mating success. Besides the ability of individuals to have many sperm to transfer to their partner, sperm transfer success is expected to depend on the interactions between the ejaculates of different donors and on the interactions between the ejaculates and the female reproductive tract [9, 75–77], both of which are potentially influenced by SFPs present in the ejaculate. Fertilization success could thus strongly depend upon the amount and composition of seminal fluid transferred.

Although the specific proteins found in the seminal fluid of *Macrostomum* have not yet been investigated directly, two recent RNA-Seq datasets provide a basis for investigating the seminal fluid composition of *M. lignano*. Firstly, Arbore et al. [78] examined differential gene expression in the head-, testis-, ovary- and tail-region of the worms. Secondly, Ramm et al. (Ramm et al: Sex Allocation Plasticity on a Transcriptome Scale: Socially-Sensitive Gene Expression in the Hermaphroditic Flatworm *Macrostomum lignano*, submitted) characterized phenotypic plasticity in *M. lignano* gene expression. Combining information from these two studies, we here aimed to perform a comprehensive whole mount ISH screen (and associated bioinformatics analyses) of 150 transcripts that are both putatively tail-specific and exhibit plastic expression in different social environments, making these strong candidates for prostate-specific expression (for detailed description of candidate selection see methods section). This strategy enabled us to identify a set of 76 putative seminal fluid transcripts.

## Methods

### Study organism

The free-living flatworm *Macrostomum lignano* is an outcrossing simultaneous hermaphrodite found in the Northern Adriatic Sea and the Eastern Mediterranean [58, 79]. As adults, the worms reach 1.5 mm in body length and the paired male and female gonads lay along the body axis on both sides of a central gut (Fig. 1). The male and female genital organs are located in the posterior part of the worms, and the former comprises the seminal vesicle (where sperm are stored prior to ejaculation), the prostate gland cells (where seminal fluid is produced) and the copulatory stylet (penis) [80]. The transparency of the worms permits the observation of internal organs and processes in vivo [58]. The worms are kept in cultures in glass petri dishes filled with artificial sea water (32‰) or nutrient-enriched artificial seawater (Guillard’s f/2 medium) [81] and fed with diatoms (*Nitzschia curvilineata*). They are kept under a 14:10 light:dark cycle at 60% relative humidity and a constant temperature of 20 °C. All the animals used in this experiment were adult worms coming from stock cultures kept either at the University of Innsbruck or the Bielefeld University and originated from the highly inbred DV1 line [65] that was also used to generate the positional RNA-Seq data [78], the socially-sensitive RNA-Seq data (Ramm et al: Sex Allocation Plasticity on a Transcriptome Scale: Socially-Sensitive Gene Expression in the Hermaphroditic Flatworm *Macrostomum lignano*, submitted), the ML2 genome assembly [59], and the recently published Mlig_3_7 genome and Mlig_RNA_3_7_DV1 transcriptome assembly [82]. The sequencing reads from both the positional and the socially-sensitive RNA-Seq data described above were mapped to the *M. lignano* de novo transcriptome assembly MLRNA110815 [78] (available online at http://www.macgenome.org/download/MLRNA110815).

### Selection of candidates

The selection of candidates for the screening for putative seminal fluid proteins is based on two recent transcriptomic datasets. Firstly, Arbore et al. [78] examined differential gene expression in the head-, testis-, ovary- and tail-region of the worms using a ‘positional’ RNA-Seq approach. To do so, they cut worms into fragments containing either (a) only the head-region, (b) the head- and testis-regions, (c) the head-, testis- and ovary-regions, or (d) the whole worm (containing all regions).. The transcripts were then assigned, according to differences in their expression pattern between fragments, to putative tissue-specific classes. Of most relevance here, a total of 366 transcripts were identified as increasing substantially (defined as a log_2_ expression fold-change > 2) in expression in the only fragment containing the tail of the worm compared to the fragment containing the head-, testis- and ovary-regions. This expression pattern makes these transcripts promising candidates to be specific to the posteriorly-located prostate gland cells (as well as other tail-specific structures). Moreover, for three of those transcripts, follow-up assays with whole mount in situ hybridization (ISH) and RNA interference (RNAi) confirmed that these were indeed specifically expressed in the prostate gland cells, but they did not show evident RNAi phenotypes when observed in vivo [78]. However, for one transcript, namely RNA815_80.4, a phenotypic effect of the RNAi treatment was evident in that the knock-down worms no longer showed labeling with the prostate-specific antibody MPr-1 [83]. This could indicate that RNA815_80.4 codes for protein containing the epitope of MPr-1 but, as Arbore et al. [78] already pointed out, it is also possible that the knock-down disrupted transcripts that are expressed earlier in the same pathway ultimately resulting in the synthesis of the protein containing the antibody’s epitope.

The second RNA-Seq study by Ramm et al. (Ramm et al: Sex Allocation Plasticity on a Transcriptome Scale: Socially-Sensitive Gene Expression in the Hermaphroditic Flatworm *Macrostomum lignano*, submitted) further refined the available expression information by characterizing phenotypic plasticity in *M. lignano* gene expression. They allocated worms to four different treatment groups, each representing different social environments favouring different optimal sex allocation patterns. They were then able to identify which transcripts are differentially expressed between these social environments (a total of ca. 10–20% of all transcripts). Of particular interest for the current study are those transcripts that are both tail-specific based on the positional classification of Arbore et al. [78] (i.e. 366 transcripts) and differentially expressed between the two most extreme social environments (worms kept isolated vs. in groups of eight) studied by Ramm et al. (Ramm et al: Sex Allocation Plasticity on a Transcriptome Scale: Socially-Sensitive Gene Expression in the Hermaphroditic Flatworm *Macrostomum lignano*, submitted). These selection criteria yielded 150 differentially expressed transcripts (i.e. 41% of the 366 transcripts), 140 of which exhibited significantly higher expression in octets than isolated individuals, with the remaining 10 showing significantly lower expression. The former is exactly the kind of phenotypically plastic expression pattern expected if these transcripts represent an aspect of male allocation in this species, making them the most obvious candidates for prostate-specific components of seminal fluid. Indeed, all three prostate-specific transcripts found by Arbore et al. [78] were also found to be differentially expressed in worms from different social group sizes in the study of Ramm et al. (Ramm et al: Sex Allocation Plasticity on a Transcriptome Scale: Socially-Sensitive Gene Expression in the Hermaphroditic Flatworm *Macrostomum lignano*, submitted). However, besides the prostate gland cells, the tail region is also the location of the developing eggs, the adhesive organs and the female and other male reproductive organs (seminal vesicle, copulatory stylet). In order to identify which of the differentially expressed tail-specific candidates are truly putative seminal fluid components, we therefore needed to refine our picture of their expression by ascertaining which are specifically and uniquely expressed in the prostate gland cells.

### Experimental rationale

The identification of the putative seminal fluid proteins is based on performing an ISH screen of selected transcripts. The transcripts were selected by combining information from both the ‘positional’ [78] and ‘social’ (Ramm et al: Sex Allocation Plasticity on a Transcriptome Scale: Socially-Sensitive Gene Expression in the Hermaphroditic Flatworm* Macrostomum lignano*, submitted) RNA-Seq data described in the previous section. Specifically, our screening effort was targeted at the 140 putative tail-specific transcripts that show a significantly higher expression in octets compared to isolated individuals, as well as the 10 that show lower expression.

### Whole-mount in situ hybridization screening

Of the 150 differentially expressed and tail-specific transcripts mentioned in the previous section, 146 were selected for the ISH screen. Four transcripts were excluded because of their low read number (< 30) in the differential expression analysis (Ramm et al: Sex Allocation Plasticity on a Transcriptome Scale: Socially-Sensitive Gene Expression in the Hermaphroditic Flatworm *Macrostomum lignano*, submitted). Forward and T7-reverse primer pairs were designed for each candidate transcript with Primer3 [84, 85] to obtain an optimal probe length of about 700 bp. cDNA was synthesized from total RNA extracted from 50 to 250 adult worms from a mass culture, using the peqGOLD cDNA Synthesis Kit H Plus (Peqlab). For probe synthesis, the cDNA was amplified with the specific primer pairs for the transcript (PCR conditions: 95 °C 5 min, (95 °C 30 s, between 55 °C and 62 °C 30 s, 72 °C 1 min 30s) × 32, 72 °C 7 min). After quality and size check by gel electrophoresis, the PCR products were purified with the Wizard SV Gel and PCR Clean-Up System Kit (Promega) and the purified products were used to synthesize single stranded anti-sense DIG-labeled RNA probes with the DIG RNA Labelling Kit (Roche). ISH was performed according to Lengerer et al. [86], using ca. fifteen adult animals in each reaction. The signal was developed at 37 °C using the NBT/BCIP system (Roche). Processed specimens were mounted in Gelvatol medium, for bright field or differential interference contrast visualization. Specimens were examined with a Leica DM5000 or an Olympus BX50. Images were taken with a Leica DFC495 digital camera and Leica LAS software or a Canon EOS 600D digital camera and Zoom Browser EX version 6.9.0a software.

### Bioinformatics analysis

For downstream bioinformatics analyses, we focused on only those transcripts that we found to be exclusively expressed in the prostate gland cells – i.e. prostate-limited transcripts. This is a subset (*n* = 76, see Results) of all prostate-specific transcripts, the latter also including transcripts that besides expression in the prostate gland cells also exhibited expression in additional tissues located elsewhere. Gene Ontology (GO) categorization of the functional annotations of the top BLASTx hits (E-value cutoff = 1e-3) was performed using the program Blast2GO [87, 88]. Gene ontology enrichment analysis was performed with Blast2GO mapping to determine protein functions in biological processes.

For all 76 transcripts, all six putative open reading frames beginning with a start codon were generated in ORF Finder (http://www.ncbi.nlm.nih.gov/gorf/gorf.html) and all resulting amino acid sequences were blasted using the Blastp Nucleotide selection (nr/nt) database at the National Center for Biotechnology Information (NCBI). Additionally, the reading frame of the nucleotide sequences was identified via the Blastx Non-redundant protein sequences (nr) database. The right ORF was identified, in some cases by blasting against the *M. lignano* genome [59], and the translated amino acid sequences were then used to test for the presence of a predicted secretory signal peptide with SignalP v4.1 [89] or for indicators of non-classical secretion inferred via SecretomeP v2.0 [90]. Additionally, the localization of the protein was predicted with the program ProtComp v9.0 [91, 92] and we checked for a predicted transmembrane helix with the program TMHMM v2.0 [93, 94].

## Results

### Identification of transcripts with prostate-specific expression

**Fig. 2:**
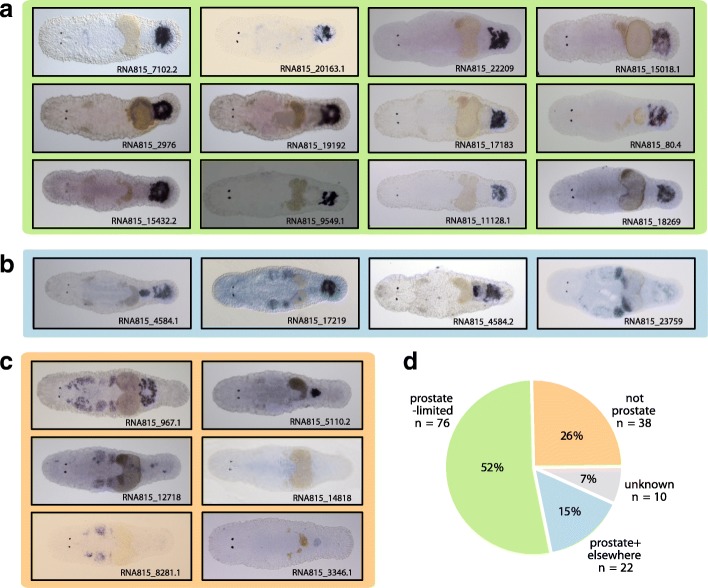
Representative whole-mount in situ hybridization expression patterns found for transcripts in *Macrostomum lignano* that are both phenotypically plastic (sensu ‘social’ RNA-Seq) and tail-limited (sensu ‘positional’ RNA-Seq). Of the 146 transcripts investigated, we obtained tissue-specific expression patterns (visualized by ISH) that can be divided into three categories, namely (**a**) prostate-limited expression; (**b**) prostate-specific expression coupled with tissue-specific expression elsewhere in the worm; and (**c**) tissue-specific expression that did not include the prostate (note that the expression pattern of 10 transcripts could not be established). Note that the classification of the observed expression pattern is based on numerous images of multiple specimens per transcript and not only on the single image shown in this figure (single images can show a somewhat misleading expression pattern because of an overstaining of the specimens or an incomplete discolourisation after the staining process). The ISH patterns depicted here in the main text are for the most highly expressed transcripts in each category; a complete catalogue of ISH images for all 136 transcripts for which we obtained a tissue-specific expression pattern is given in Additional file 1: Figure S1-S3 and the total number of transcripts in each category are given in (**d**)

Our goal was to screen the candidate transcripts for the prostate-limited expression expected of seminal fluid proteins. We performed ISH for 146 transcripts, to ascertain the site(s) of their expression. Overall, 76 of the 146 tested transcripts show a prostate-limited expression (Fig. 2a, and Additional file 1: Figure S1), indicating that more than half of our transcripts encode potential seminal fluid proteins. A further 22 transcripts exhibit specific expression in the prostate gland cells, but are also expressed in other tissues (Fig. 2b, and Additional file 1: Figure S2), with 50% also exhibiting expression in the gonads (mostly testis, but also ovary, or both) and 15% in the pharyngeal glands. Additionally, 38 transcripts are not expressed in the prostate (Fig. 2c, and Additional file 1: Figure S3); the expression pattern of the remaining 10 transcripts remains unknown, since no pattern could be found with ISH or it was not possible to amplify these transcripts by PCR and therefore a hindrance for probe synthesis and subsequent ISH assays. Overall, 67% of the tested transcripts therefore show either expression in the prostate exclusively or in the prostate and other tissue combined (Fig. 2d). We note here that three of the ‘non-prostate’ transcripts (815_10124.3, 815_35136 and 815_97.1) were found to have specific expression in the adhesive glands, as do several of the tail-specific transcripts that are not plastically expressed in different social environments, as reported elsewhere [95].

**Fig. 3:**
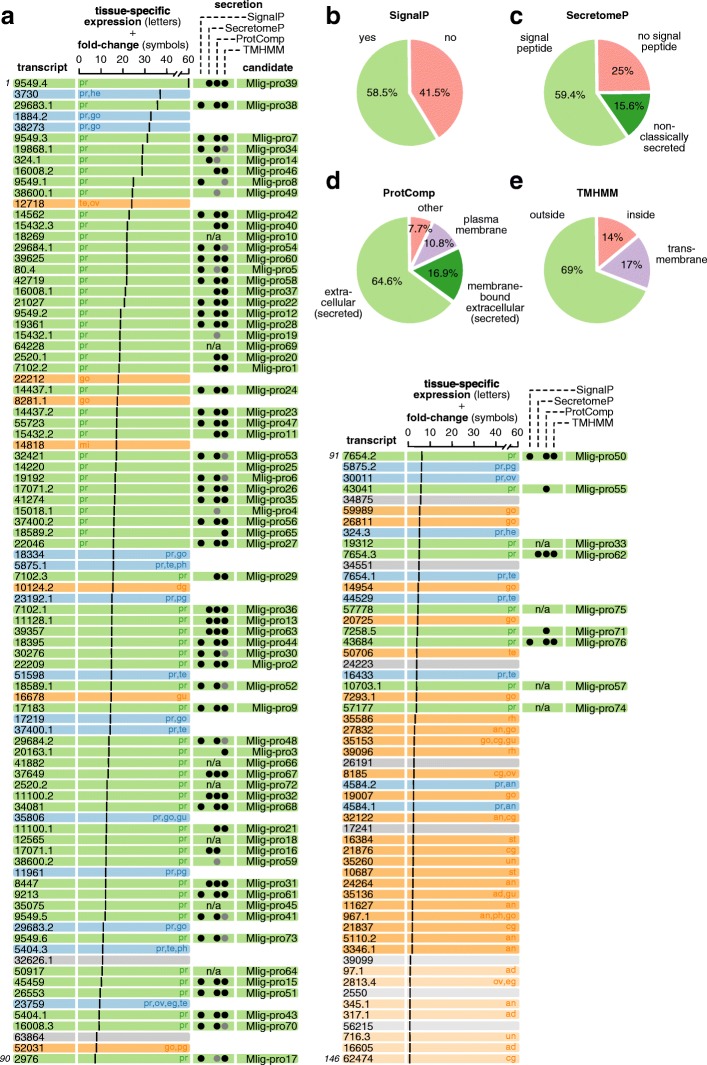
Summary of the expression patterns (visually identified by ISH) for all 146 tail-specific transcripts investigated and secretion predictions for those 76 with prostate-limited expression. In (**a**) the transcripts are listed in descending order of their fold-change (= solid vertical bar) observed in an earlier study comparing transcript expression in octets (groups of eight worms) compared to isolated worms (Ramm et al: Sex Allocation Plasticity on a Transcriptome Scale: Socially-Sensitive Gene Expression in the Hermaphroditic Flatworm *Macrostomum lignano*, submitted). Note that the 10 transcripts at the end of the list have fold-change values less than one (i.e. they had lower expression in octets than in isolated worms). For each transcript, we document its site of expression, indicated by the colour of the bar (green: prostate-limited; blue: prostate + elsewhere; orange: not prostate; grey: unknown) and by the abbreviated tissue assignments (pr: prostate; he: head; te: testis; ov: ovary; go: gonad; mi: unspecific in middle section; ph: pharynx; pg: pharyngeal glands; gu: gut; eg: egg; rh: rhabdites; an: antrum; cg: cement glands; st: stylet; un: unspecific; ad: adhesive glands). In addition, for each of the 76 prostate-limited transcripts we assign a unique number (Mlig-pro1–76, see main text for explanation) and sought bioinformatic evidence for secretion. For the 50 transcripts for which we could perform bioinformatic analyses, we indicate positive evidence for secretion based on predictions from SignalP (black circle: signal peptide present); SecretomeP (black circle: non-classically secreted); ProtComp (black circle: extra-cellularly secreted or membrane-bound extracellularly secreted; grey circle: plasma membrane); and TMHMM (black circle: outside the cell; grey circle: trans-membrane helix). Note that for 11 transcripts, it was not possible to identify the open reading frame and bioinformatics analyses could therefore not be performed (denoted ‘n/a’). The results from each of these analyses are summarized in panels (**b**-**e**)

With this information on tissue-specificity at hand, we can now revisit the gene expression data that was used to identify candidates. Doing so reveals a striking pattern: as expected, phenotypically plastic expression represents a signature of prostate-specificity, confirming our original rationale. It is indeed those genes which showed the most plastic expression in the social RNA-Seq study (Ramm et al: Sex Allocation Plasticity on a Transcriptome Scale: Socially-Sensitive Gene Expression in the Hermaphroditic Flatworm *Macrostomum lignano*, submitted) that were most likely to be identified as prostate-limited or prostate-specific in our ISH screen (Fig. 3a). Based on this strong agreement between the independently derived RNA-Seq and ISH gene expression data, we therefore consider this subset of 76 transcripts with plastic, prostate-limited expression as putative prostate-specific transcripts, naming them Mlig-pro1 through Mlig-pro76 (i.e. *Macrostomum* *lignano* prostate transcripts 1–76), and numbering them in descending order of their overall expression level in octets in the social RNA-Seq study (Ramm et al: Sex Allocation Plasticity on a Transcriptome Scale: Socially-Sensitive Gene Expression in the Hermaphroditic Flatworm *Macrostomum lignano*, submitted) (Fig. 3a).

### Prediction of protein secretion and localization

To predict whether or not the 76 identified transcripts that exhibited prostate-limited expression in our ISH screen are likely to code for seminal fluid proteins that are transferred to the mating partner – or alternatively for prostate-limited proteins that are not secretory, presumably meaning they are some part of the seminal fluid production machinery within the prostate gland cells – we sought evidence for secretion and final location using bioinformatics tools. Specifically, the translated amino acid sequences of each SFP candidate transcript were analyzed using SignalP to detect predicted signal peptides associated with classical secretion [89]. Additionally, candidate transcripts were analyzed with SecretomeP to detect other motifs for secretion that are associated with non-classical secretory pathways [96]. Because we expect that seminal fluid proteins tend to be extracellularly secreted proteins, they can be expected to have a signal peptide [6, 28].

In total, we could predict the putative ORF for 65 of the 76 candidate transcripts and translated it into the amino acid sequence. All of these 65 translated transcripts start with a start codon and, except for 6 candidates, stop at a stop codon. For the remaining 11 transcripts, it was not possible to identify the ORF.

According to analysis using SignalP, 38 of the 65 identified proteins with prostate-limited expression were predicted to be secreted by the signal peptide associated with the classical secretory pathway (Fig. 3a, b). Of the 27 remaining proteins without such a signal peptide, 10 were predicted to be secreted via a non-classical pathway according to analysis using SecretomeP (Fig. 3a, c). Note that one of the transcripts had to be excluded from that analysis because its translated sequence was shorter than the minimally required 40 amino acids needed for a SecretomeP analysis. So in total, 48 of the analyzed 65 prostate-limited proteins (74%) show evidence for secretion, making these especially strong candidates as putative seminal fluid proteins transferred during mating. However, we note that this could be an underestimate, because for some proteins it is possible that a signal peptide was not detected, not because it is truly absent, but because we do not know the full protein sequence. We note, however, that it is also possible that we currently overestimate the total number of SFPs, because some of the transcript fragments in the current transcriptome assembly could ultimately prove to belong to the same protein.

The predicted protein location was analyzed by ProtComp to find candidates that are localized as ‘extracellular’ and/or have a ‘plasma membrane’ destination. Of the candidates, 42 are predicted to be extracellularly secreted, 11 are membrane-bound extracellular and 7 are a part of the plasma membrane (Fig. 3a, d).

Since the signal peptide can be recognized as a membrane helix [97], TMHMM searches were also performed to predict the cellular location of the protein as being either outside the cell, inside the cell or trans-membrane. Eleven of the candidates had a membrane helix, nine were predicted to be located inside of the membrane and 45 to be outside (Fig. 3e).

Summing up these different bioinformatics analyses, we posit that candidates are likely to be seminal fluid proteins if they show at least one of the following characteristics: (a) a predicted signal peptide inferred via SignalP or a motif associated with a non-classical secretory pathway inferred from SecretomeP; (b) localization as extracellular and/or with plasma membrane destination inferred via ProtComp; or (c) the recognition of a membrane helix inferred via TMHMM. Almost all candidates analysed fulfil at least one of these criteria: a total of 64 candidates (of 65 analysed) therefore currently are our top candidates to be seminal fluid proteins, though we note that the transcripts that were not analyzed, be it because they were shorter than 40AA or because we could not identify the ORF, should also not be excluded as putative candidates.

### Gene ontology classification

**Fig. 4:**
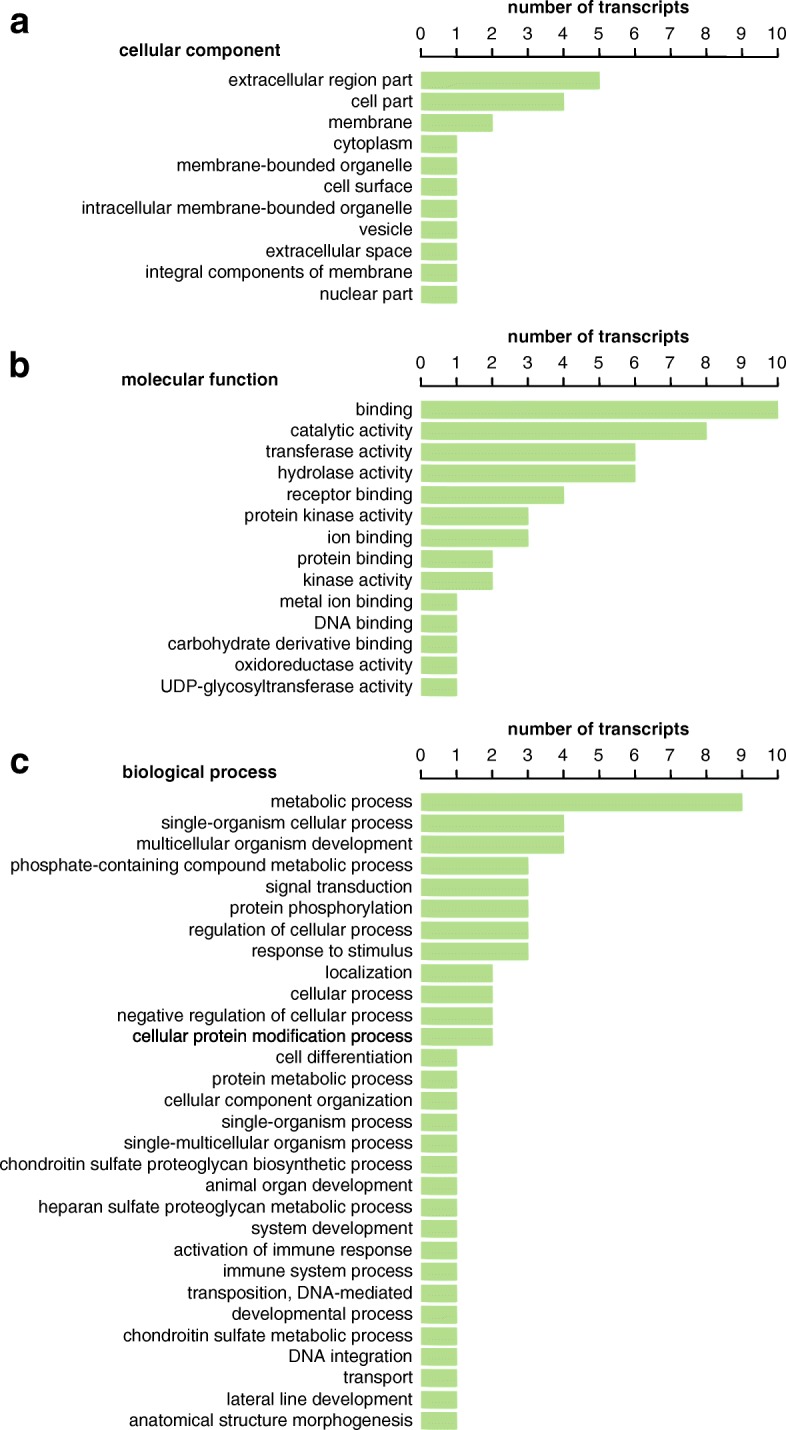
Summary of Gene Ontology (GO) analysis using Blast2GO of 76 prostate-limited transcripts. **a** GO term distribution for the cellular component domain; (**b**) GO term distribution for the molecular function domain; and (**c**) GO term distribution for the biological processes domain

To characterize the likely functions of our seminal fluid candidates, we obtained Gene Ontology (GO) classifications for the 76 transcripts with prostate-limited expression in three ontology domains: cellular component, molecular function and biological process. It is important to note that a transcript could be included in several different categories and be associated with multiple GO annotations within a single category. This therefore results in more GO-annotations than sequences annotated. Blast2GO revealed that 31 transcripts had no Blast hits against the non-redundant protein NCBI database. Of the remaining 45 (59%) transcripts that had a Blast hit, we overall could assign GO annotations to 44 transcripts. An overview of the distribution of the sequences in the three ontology domains can be seen in Fig. 4. In the cellular component category (Fig. 4a), there is a clear predominance of extracellular region parts and cell parts compared to intracellular regions. Regarding the molecular function classification (Fig. 4b), the main functions are involved in binding, catalytic activity, transferase activity, hydrolase activity, receptor binding and protein kinase activity. In the biological process category (Fig. 4c), the prevailing groups are associated with metabolic processes, single-organism cellular processes and multicellular organism development. With a total number of 6 each, the most often identified proteins were fungistatic metabolites or transmembrane receptors (For a more detailed description of the sequences and the BLAST hits see Additional file 2: Table S1).

## Discussion

Using a combination of transcriptomic data (Ramm et al: Sex Allocation Plasticity on a Transcriptome Scale: Socially-Sensitive Gene Expression in the Hermaphroditic Flatworm *Macrostomum lignano*, submitted) [78] and ISH experiments (present study), we are able to identify 76 transcripts in *M. lignano* which show specific expression that is limited to the prostate, as expected for SFPs. The identification of a large number of SFP candidates is in line with similarly large numbers of identified proteins in other species studied to date (e.g. 198 SFPs in *Aedes albopictus* [24], more than 200 in *Drosophila melanogaster* [13], 69 in *Mus domesticus* [36] and several hundred in humans [40, 41]), supporting the notion that seminal fluid is a complex and diverse secretion [3, 35]. We consider it less likely that the 22 transcripts that are expressed in the prostate and additionally in other tissues are potential seminal fluid candidates, because we expect seminal fluid proteins to be exclusively expressed in the prostate (or in other accessory male reproductive organs, depending on the taxon). Nevertheless, these are also of interest in the current context, because they could play important roles in seminal fluid production. By far the most prevalent ‘mixed’ expression pattern was for expression in the prostate and the testis, suggesting a function specific to the male sex function, though the precise significance of this co-expression remains to be determined. In fact, we cannot definitely exclude that transcripts with such a mixed expression in the prostate gland cells and the testis are also transferred SFPs, because there is the possibility that these proteins pass from the testis through the vas deferens together with the sperm.

Several of the transcripts had no identifiable homologues in Blast searches and had also no conserved protein domains, indicating that these proteins are likely highly diverged compared to previously characterized proteins. This is perhaps due both to the relatively distant phylogenetic position of our study organism with respect to previously studied taxa, and/or because the proteins themselves are rapidly evolving. Again, our data are consistent with the fact that many SFP sequences are thought to evolve rapidly due to the fact that they are a target of (sexual) selection [6–11, 14] and sexual conflict [13, 42].

Many previous studies seeking to identify seminal fluid proteins have used proteomics approaches (e.g. [17, 35, 36, 98–102]), but because of the small size of *M. lignano*, collecting proteins from ejaculates is currently challenging. To overcome this limitation, we employed an alternative approach to identify putative SFPs. Specifically, we prioritized transcripts as seminal fluid protein candidates based on three assumptions, namely (i) that they are more highly expressed in larger groups, reflecting a transcriptional upregulation in response to the high level of sperm competition experienced at larger group size compared to the non-mating environment when isolated (a phenotypically plastic response that has been demonstrated also in other taxa [103–105]; (ii) that they show an expression limited to the prostate glands; and (iii) that they exhibit positive evidence of being secreted. Of the 65 prostate-limited transcripts investigated for signs of secretion and their location in the cell, 74% were predicted to be secreted either via a signal pathway or by a non-classical secretory pathway. This is a much higher percentage than would be predicted for the whole proteome – in humans, for example, only between 10 and 20% of all proteins are secreted [106] – but because we were looking for proteins that are part of the ejaculate and therefore are transferred to the mating partner, this is in line with our expectations. This result is also in concordance with the fact that 69% of the proteins we found by TMHMM analysis were located outside of the cell and that 82% of the proteins were predicted to be either extracellularly secreted or to be extracellularly membrane-bound. In total, 64 of the 65 candidates fulfill our criteria for secretion (secretion, transmembrane, part of plasma membrane or extracellularly located), providing strong indirect support for their assignment as putative SFPs and a clear vindication of our identification strategy.

We note also that this list of putative SFPs is likely to be an underestimate, since 11 transcripts with confirmed prostate-limited expression could not be analyzed, due to the absence of conserved protein domains and homology by Blast search. As already mentioned in the Results section, this conclusion comes with one important proviso, namely that there is also potentially some redundancy in our candidate list, in that some of the different transcript fragments tested could in fact belong to the same protein or represent different protein isoforms produced by alternative splicing. Especially the transcripts with the same main number that end with different sub-numbers (e.g. 29684.1 and 29684.2) are likely to belong to the same protein or different isoforms of that protein. They show a high sequence similarity with each other and when we blasted them against the *M. lignano* genome (ML2 assembly) [59] they always align to the same region within the same contig.

To begin to characterize potential functions of the putative seminal fluid proteins, we used Blast2Go to classify them according to their predicted molecular functions, involvement in biological processes, and cellular components. However, the fact that many of the functional categories are extremely broad (such as ‘binding’ or ‘catalytic activity’), and that the same putative proteins can be assigned to several categories, makes interpreting these classifications far from straightforward. Nevertheless, the fact that in the cellular components classification the biggest fraction belongs to the category “extracellular region part” is in concordance with the findings just discussed before that the majority is extracellularly secreted.

To move beyond these broad functional classifications, we need to directly assess the roles of specific SFPs. The next steps are therefore to evaluate which of these SFP candidates are actually transferred to the mating partner during insemination, and especially to elucidate what effect they have on the mating partner and the reproductive success of the sperm donor. Due to the availability of applying RNAi in *Macrostomum* it is possible to knock-down the expression of specific transcripts and to test for their specific functions in mating experiments (cf. [86, 107–110]). Moreover, due to the availability of a GFP-expressing line there is also the possibility to readily assign paternity following double-mating experiments. In this way, we can examine the fitness consequences of knocking down SFP expression, to test directly for the ability of a donor worm, missing a specific SFP, to compete against a rival [65, 74, 111].

## Conclusions

In summary, our study represents the first large-scale screen to identify putative SFPs in a flatworm, identifying 76 transcripts in *M. lignano* with prostate-limited expression. Of these, at least 64 also exhibit evidence of being secreted and therefore of being transferred SFPs. These putative SFPs are now exciting candidates for future genetic and behavioral studies to examine the function of this important class of proteins.

## Additional files

Additional file 1: **Figure S1-S3.** Whole-mount in situ hybridization expression patterns found for transcripts in *Macrostomum lignano* that are both phenotypically plastic (sensu ‘social’ RNA-Seq) and tail-limited (sensu ‘positional’ RNA-Seq). Of the 146 transcripts investigated, we obtained tissue-specific expression patterns that can be divided into three categories, namely (**Figure S1.**) prostate-limited expression; (**Figure S2.**) prostate-specific expression coupled with tissue-specific expression elsewhere in the worm; and (**Figure S3.**) tissue-specific expression that did not include the prostate. The expression patterns of 10 transcripts could not be established. Within each category, pictures are arranged (from top-left to bottom-right) in descending order of fold-change in expression in octets versus isolated worms. One representative picture per transcript is included. (ZIP 5699 kb)

Additional file 2: **Table S1.** Summary of top blast hits identified by Blast2Go. (PDF 55 kb)

## Abbreviations

ISH: Whole-mount in situ hybridization
SFP: Seminal fluid protein
