# Supplementary figures and images for: A targeted in situ hybridization screen identifies putative seminal fluid proteins in a simultaneously hermaphroditic flatworm
Source: BMC Evol Biol. 2018 May 30;18:81. doi: 10.1186/s12862-018-1187-0 (PMC5977470; doi:10.1186/s12862-018-1187-0)

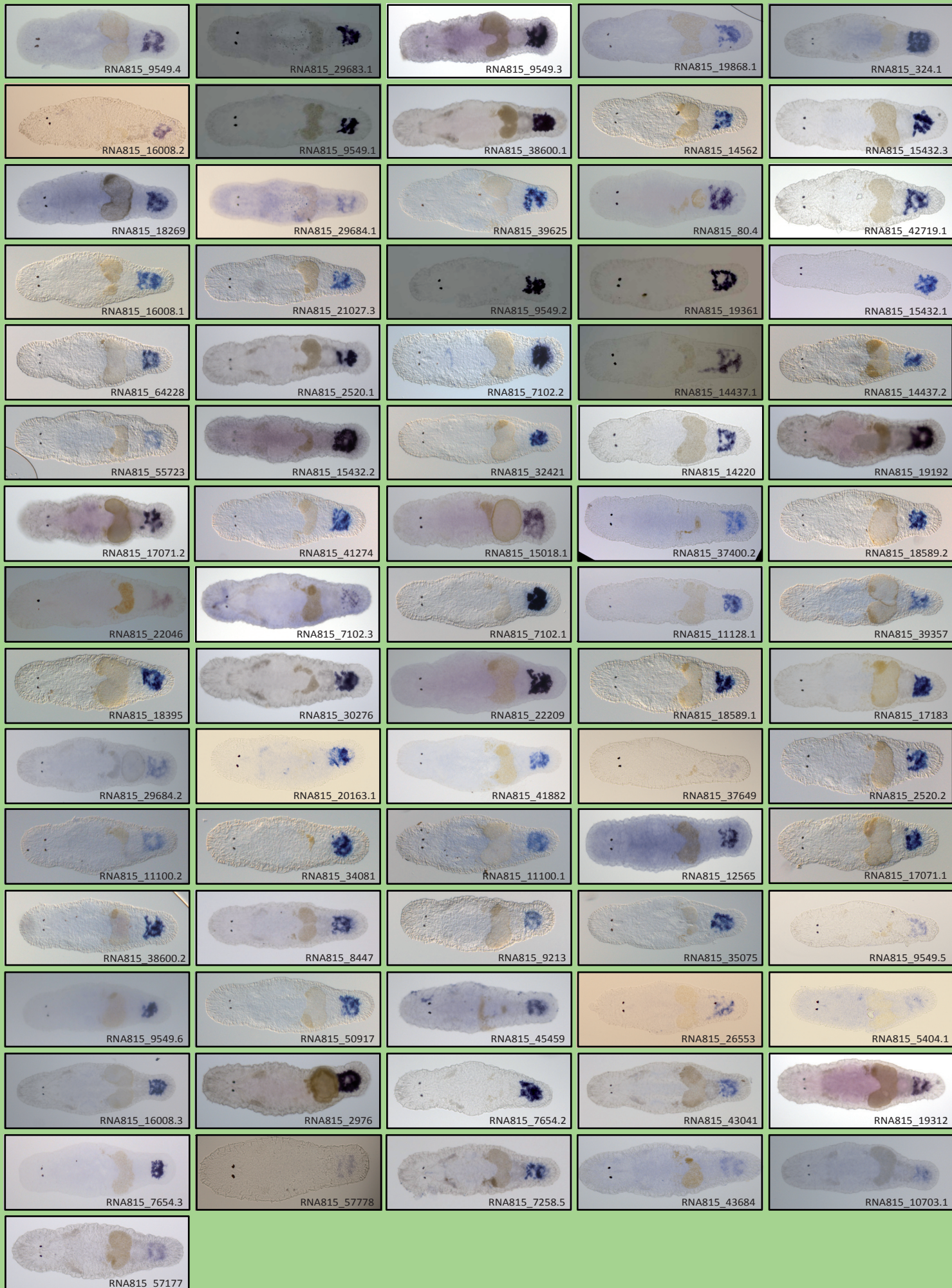

Supplement: Supplementary file 1 — Figure S1-S3. Whole-mount in situ hybridization expression patterns found for transcripts in Macrostomum lignano that are both phenotypically plastic (sensu ‘social’ RNA-Seq) and tail-limited (sensu ‘positional’ RNA-Seq). Of the 146 transcripts investigated, we obtained tissue-specific expression patterns that can be divided into three categories, namely (Figure S1.) prostate-limited expression; (Figure S2.) prostate-specific expression coupled with tissue-specific expression elsewhere in the worm; and (Figure S3.) tissue-specific expression that did not include the prostate. The expression patterns of 10 transcripts could not be established. Within each category, pictures are arranged (from top-left to bottom-right) in descending order of fold-change in expression in octets versus isolated worms. One representative picture per transcript is included. (ZIP 5699 kb) [file 12862_2018_1187_MOESM1_ESM.zip › Fig_S1.pdf]

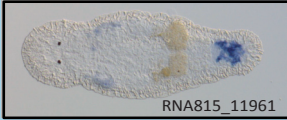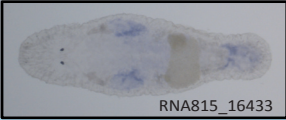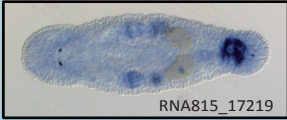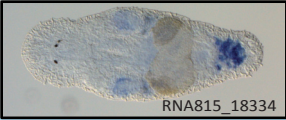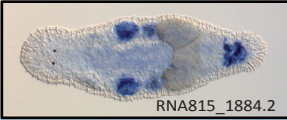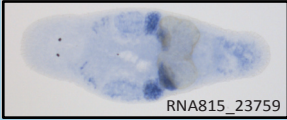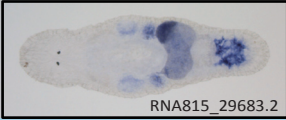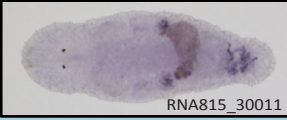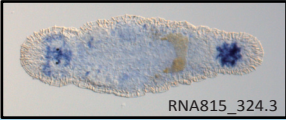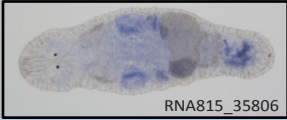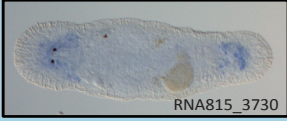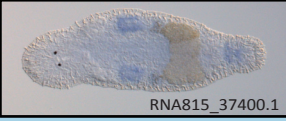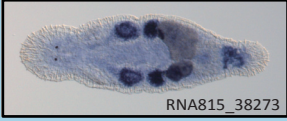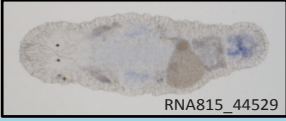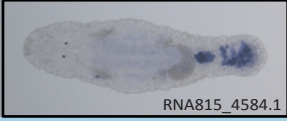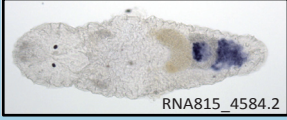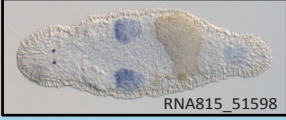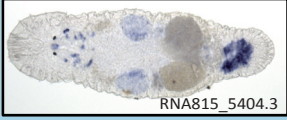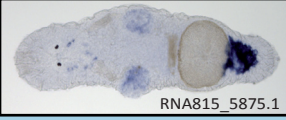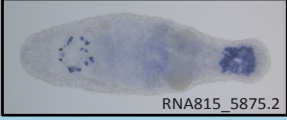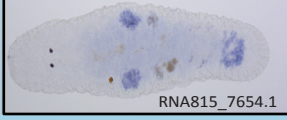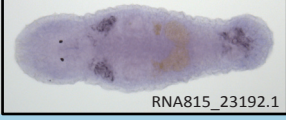

Supplement: Supplementary file 1 — Figure S1-S3. Whole-mount in situ hybridization expression patterns found for transcripts in Macrostomum lignano that are both phenotypically plastic (sensu ‘social’ RNA-Seq) and tail-limited (sensu ‘positional’ RNA-Seq). Of the 146 transcripts investigated, we obtained tissue-specific expression patterns that can be divided into three categories, namely (Figure S1.) prostate-limited expression; (Figure S2.) prostate-specific expression coupled with tissue-specific expression elsewhere in the worm; and (Figure S3.) tissue-specific expression that did not include the prostate. The expression patterns of 10 transcripts could not be established. Within each category, pictures are arranged (from top-left to bottom-right) in descending order of fold-change in expression in octets versus isolated worms. One representative picture per transcript is included. (ZIP 5699 kb) [file 12862_2018_1187_MOESM1_ESM.zip › Fig_S2.pdf]

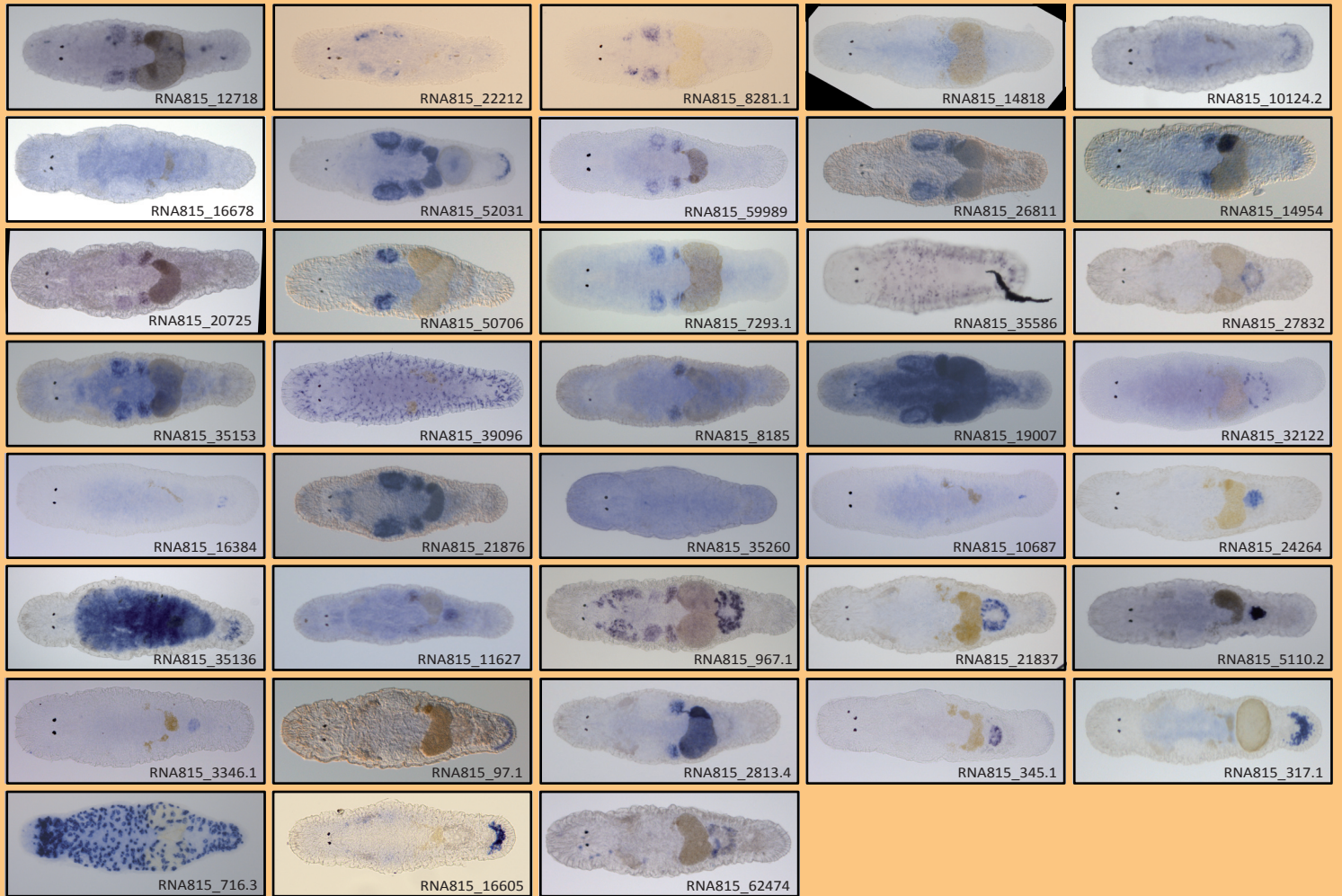

Supplement: Supplementary file 1 — Figure S1-S3. Whole-mount in situ hybridization expression patterns found for transcripts in Macrostomum lignano that are both phenotypically plastic (sensu ‘social’ RNA-Seq) and tail-limited (sensu ‘positional’ RNA-Seq). Of the 146 transcripts investigated, we obtained tissue-specific expression patterns that can be divided into three categories, namely (Figure S1.) prostate-limited expression; (Figure S2.) prostate-specific expression coupled with tissue-specific expression elsewhere in the worm; and (Figure S3.) tissue-specific expression that did not include the prostate. The expression patterns of 10 transcripts could not be established. Within each category, pictures are arranged (from top-left to bottom-right) in descending order of fold-change in expression in octets versus isolated worms. One representative picture per transcript is included. (ZIP 5699 kb) [file 12862_2018_1187_MOESM1_ESM.zip › Fig_S3.pdf]
