# Supplementary material for: A targeted in situ hybridization screen identifies putative seminal fluid proteins in a simultaneously hermaphroditic flatworm
Source: BMC Evol Biol. 2018 May 30;18:81. doi: 10.1186/s12862-018-1187-0 (PMC5977470; doi:10.1186/s12862-018-1187-0)
Supplement: Supplementary file 2 — Table S1. Summary of top blast hits identified by Blast2Go. (PDF 55 kb) [file 12862_2018_1187_MOESM2_ESM.pdf]

Sequence name	Sequence desc.	Hit desc.	Hit ACC	E-Value	Similarity	Sequence length	Alignment length	Positives
RNA815_10703.1	---NA---	No Blast Hit				1389		
RNA815_11100.2	glucan endo-1,3-alpha-glucosidase agn1	hypothetical protein MYCFIDRAFT_128488, partial [Cercospora fijiensis CIRAD86]	XP_007922170, EME89605	8.11E-14	48.50	1183	167	81
RNA815_11100.1	glucan endo-1,3-alpha-glucosidase agn1	PREDICTED: uncharacterized protein LOC105345065 [Crassostrea gigas]	XP_011451345	3.23E-16	48.74	1358	199	97
RNA815_11128.1	angiopoietin-related 1	PREDICTED: microfibril-associated glycoprotein 4-like [Clupea harengus]	XP_012672607	1.13E-20	57.86	1356	140	81
RNA815_12565	---NA---	No Blast Hit				1255		
RNA815_14220	ficolin-2-like isoform X2	PREDICTED: ficolin-1-like isoform X1 [Dipodomys ordii]	XP_012884848	1.38E-15	47.66	1152	128	61
RNA815_14437.2	Galactose beta- partial	putative fungistatic metabolite [Escovopsis weberi]	KOS19608	1.41E-10	39.66	1044	232	92
RNA815_14437.1	fungistatic metabolite	putative fungistatic metabolite [Escovopsis weberi]	KOS19608	6.35E-10	39.91	1140	233	93
RNA815_14562	GCC2 and GCC3 domain containing	protein kinase, putative (ISS) [Galdieria sulphuraria]	XP_005703766, EME27246	2.02E-22	53.80	1133	184	99
RNA815_15018.1	WSC-domain-containing [Auricularia subglabra TFB-10046 SS5]	WSC-domain-containing protein [Auricularia subglabra TFB-10046 SS5]	XP_007337249, EJD54381	3.54E-18	45.83	1111	216	99
RNA815_15432.3	beta-1,6-N- contains WSC domain	beta-1,6-N-acetylglucosaminyltransferase, contains WSC domain [Xanthophyllomyces dendrorhous]	CDZ98531	1.35E-05	44.07	706	118	52
RNA815_15432.2	transmembrane receptor	hypothetical protein S40285_06647 [Stachybotrys chlorohalonata IBT 40285]	KFA68284	1.66E-09	58.97	981	78	46
RNA815_15432.1	WSC-domain-containing [Auricularia subglabra TFB-10046 SS5]	beta-1,6-N-acetylglucosaminyltransferase, contains WSC domain [Xanthophyllomyces dendrorhous]	CDZ98531	2.86E-15	44.86	1089	214	96
RNA815_16008.3	WSC-domain-containing [Aureobasidium namibiae CBS]	PREDICTED: uncharacterized protein LOC105345065 [Crassostrea gigas]	XP_011451345	3.38E-10	55.17	264	87	48
RNA815_16008.2	fungistatic metabolite	hypothetical protein TREMEDRAFT_38501 [Tremella mesenterica DSM 1558]	XP_007003930, EIW69737	5.92E-14	49.70	555	165	82
RNA815_16008.1	fungistatic metabolite	PREDICTED: putative fungistatic metabolite [Crassostrea gigas]	XP_011448796	1.85E-44	53.77	1062	292	157
RNA815_17071.2	WSC domain-containing 1	hypothetical protein A6R68_17596, partial [Neotoma lepida]	OBS75953	8.74E-09	52.53	693	99	52
RNA815_17071.1	transmembrane receptor	Plasminogen [Crassostrea gigas]	EKC22375	2.23E-14	48.79	1014	207	101
RNA815_17183	fungistatic metabolite	Putative fungistatic metabolite [Crassostrea gigas]	EKC22376	6.24E-13	56.76	1009	74	42
RNA815_18269	---NA---	No Blast Hit				965		
RNA815_18395	GCC2 and GCC3 domain containing	hypothetical protein, conserved [Cyanidioschyzon merolae strain 10D]	XP_005536506, BAM80470	1.06E-41	53.94	960	317	171
RNA815_18589.2	---NA---	No Blast Hit				616		
RNA815_18589.1	WSC domain-containing	hypothetical protein AOL_s00006g367 [Arthrobotrys oligospora ATCC 24927]	XP_011117978, EGX53501	9.03E-13	49.32	951	148	73
RNA815_19192	---NA---	No Blast Hit				929		
RNA815_19312	---NA---	No Blast Hit				925		

RNA815_19361	fungistatic metabolite	hypothetical protein HMPREF1541_02758 [Cyphellophora europaea CBS 101466]	XP_008715335, ETN43599	4.25E-09	42.17	923	166	70
RNA815_19868.1	Centrosomal POC5	hypothetical protein [Hippea alviniae]	WP_022670342	4.70E-17	61.80	906	89	55
RNA815_20163.1	copper radical oxidase (WSC domain-containing)	hypothetical protein V493_00321 [Pseudogymnoascus sp. VKM F-4281 (FW-2241)]	KFY32305	8.26E-21	48.10	897	210	101
RNA815_21027	beta-1,3 exoglucanase precursor	hypothetical protein V502_04028 [Pseudogymnoascus sp. VKM F-4520 (FW-2644)]	KFZ18605	1.41E-07	48.98	869	98	48
RNA815_22046	PREDICTED: uncharacterized protein LOC101863664 isoform X2	PREDICTED: uncharacterized protein LOC101863664 isoform X2 [Aplysia californica]	XP_012936700	1.98E-06	54.17	838	96	52
RNA815_22209	---NA---	No Blast Hit				833		
RNA815_2520.2	---NA---	No Blast Hit				452		
RNA815_2520.1	Paired box and Transposase domain containing	transposase [Ancylostoma ceylanicum]	EPB77486	3.08E-44	70.31	2649	64	45
RNA815_26553	---NA---	No Blast Hit				724		
RNA815_29683.1	WSC domain-containing	hypothetical protein NECHADRAFT_91549 [Nectria haematococca mpVI 77-13-4]	XP_003041594, EEU35881	4.75E-06	52.87	661	87	46
RNA815_29684.2	---NA---	No Blast Hit				486		
RNA815_29684.1	---NA---	No Blast Hit				661		
RNA815_2976	serine threonine	protein kinase, putative (ISS) [Galdieria sulphuraria]	XP_005703766, EME27246	5.10E-53	50.65	2494	385	195
RNA815_30276	glycoside hydrolase family 55	hypothetical protein V496_01885 [Pseudogymnoascus sp. VKM F-4515 (FW-2607)]	KFY66882, KFZ00287	2.27E-10	50.00	650	106	53
RNA815_324.1	hypothetical protein BRAFLDRAFT_67600	hypothetical protein BRAFLDRAFT_67600 [Branchiostoma floridae]	XP_002606357, EEN62367	1.48E-122	56.30	5161	659	371
RNA815_32421	fungistatic metabolite	Plasminogen [Crassostrea gigas]	EKC22375	2.15E-18	51.19	611	168	86
RNA815_34081	---NA---	No Blast Hit				585		
RNA815_35075	---NA---	No Blast Hit				570		
RNA815_37400.2	---NA---	No Blast Hit				417		
RNA815_37649	secretory calcium-binding phosphospho proline-glutamine-rich partial	secretory calcium-binding phosphosphoprotein proline-glutamine-rich 11, partial [Lepisosteus oculatus]	AMD08918	6.67E-09	63.11	534	122	77
RNA815_38600.2	Cyclin and Carbohydrate-binding WSC and Glycosyl transferase domain containing	Cyclin and Carbohydrate-binding WSC and Glycosyl transferase domain containing protein [Haemonchus contortus]	CDJ95165	1.18E-10	54.65	486	86	47
RNA815_38600.1	WSC-domain-containing [Auricularia subglabra TFB-10046 SS5]	conserved unknown protein [Ectocarpus siliculosus]	CBN78573	3.46E-10	51.00	522	100	51
RNA815_39357	WSC domain-containing 2	conserved unknown protein [Ectocarpus siliculosus]	CBN78573	4.98E-09	54.46	513	101	55
RNA815_39625	---NA---	No Blast Hit				510		
RNA815_41274	---NA---	No Blast Hit				492		
RNA815_41882	---NA---	No Blast Hit				486		
RNA815_42719	---NA---	No Blast Hit				478		

RNA815_43041	---NA---	No Blast Hit				475		
RNA815_43684	---NA---	No Blast Hit				469		
RNA815_45459	---NA---	No Blast Hit				454		
RNA815_50917	angiopoietin-related 7- partial	uncharacterized protein Dwil_GK24749 [Drosophila willistoni]	XP_002066950, EDW77936	4.67E-05	79.41	400	34	27
RNA815_5404.1	A disintegrin and metallo ase with thrombospondin motifs 20-like	hypothetical protein A3Q56_00734 [Intoshia linei]	OAF71492	6.79E-29	48.32	1970	327	158
RNA815_55723	---NA---	No Blast Hit				331		
RNA815_57177	---NA---	No Blast Hit				307		
RNA815_57778	---NA---	No Blast Hit				297		
RNA815_64228	serine threonine	PREDICTED: signal peptide, CUB and EGF-like domain-containing protein 3 [Biomphalaria glabrata]	XP_013088395	5.87E-11	62.69	210	67	42
RNA815_7102.3	---NA---	No Blast Hit				861		
RNA815_7102.2	transmembrane receptor	beta-1,6-N-acetylglucosaminyltransferase, contains WSC domain [Xanthophyllomyces dendrorhous]	CDZ98531	5.34E-13	45.00	936	180	81
RNA815_7102.1	transmembrane receptor	beta-1,6-N-acetylglucosaminyltransferase, contains WSC domain [Xanthophyllomyces dendrorhous]	CDZ98531	5.15E-12	52.94	1736	85	45
RNA815_7258.2	---NA---	No Blast Hit				486		
RNA815_7654.2	---NA---	No Blast Hit				354		
RNA815_7654.1	---NA---	No Blast Hit				399		
RNA815_80.4	transmembrane receptor	PREDICTED: uncharacterized protein LOC105345065 [Crassostrea gigas]	XP_011451345	1.32E-12	46.30	969	162	75
RNA815_8447	---NA---	No Blast Hit				1583		
RNA815_9213	---NA---	No Blast Hit				1512		
RNA815_9549.6	---NA---	No Blast Hit				469		
RNA815_9549.5	---NA---	No Blast Hit				563		
RNA815_9549.4	microfibril-associated glyco 4-like	PREDICTED: fibrinogen C domain-containing protein 1-like [Drosophila suzukii]	XP_016938590	3.38E-20	48.79	828	207	101
RNA815_9549.3	microfibril-associated glyco 4-like	PREDICTED: microfibril-associated glycoprotein 4-like [Larimichthys crocea]	XP_010748505	2.22E-20	53.02	1199	149	79
RNA815_9549.2	fibrinogen C domain-containing 1-like	PREDICTED: ficolin-2-like, partial [Aplysia californica]	XP_005113499	4.04E-23	53.53	1248	170	91
RNA815_9549.1	fibrinogen A	PREDICTED: ficolin-2-like, partial [Aplysia californica]	XP_005113499	2.26E-22	53.53	1480	170	91
